# Categorical perception of lexical tones in mandarin-speaking congenital amusics

**DOI:** 10.3389/fpsyg.2015.00829

**Published:** 2015-06-16

**Authors:** Wan-Ting Huang, Chang Liu, Qi Dong, Yun Nan

**Affiliations:** ^1^State Key Laboratory of Cognitive Neuroscience and Learning, IDG/McGovern Institute for Brain Research, Beijing Normal UniversityBeijing, China; ^2^Department of Communication Sciences and Disorders, The University of Texas at AustinAustin, TX, USA

**Keywords:** congenital amusia, tone agnosia, tone identification, tone discrimination, categorical perception

## Abstract

Previous research suggests that within Mandarin-speaking congenital amusics, only a subgroup has behavioral lexical tone perception impairments (tone agnosia), whereas the rest of amusics do not. The purpose of the current study was to investigate the categorical nature of lexical tone perception in Mandarin-speaking amusics with and without behavioral lexical tone deficits. Three groups of listeners (controls, pure amusics, and amusics with tone agnosia) participated in tone identification and discrimination tasks. Indexes of the categorical perception (CP) of a physical continuum of fundamental frequencies ranging from a rising to level tone were measured. Specifically, the stimulus durations were manipulated at 100 and 200 ms. For both stimulus durations, all groups exhibited similar categorical boundaries. The pure amusics showed sharp identification slopes and significantly peaked discrimination functions similar to those of normal controls. However, such essential characteristics for the CP of lexical tones were not observed in amusics with tone agnosia. An enlarged step-size from 20 to 35 Hz was not able to produce any discrimination peaks in tone agnosics either. The current study revealed that only amusics with tone agnosia showed a lack of categorical tone perception, while the pure amusics demonstrated typical CP of lexical tones, indicating that the deficit of pitch processing in music does not necessarily result in the deficit in the CP of lexical tones. The different performance between congenital amusics with and without tone agnosia provides a new perspective on the proposition of the relationship between music and speech perception.

## Introduction

Unlike most people who can enjoy music at their leisure, approximately 4% of the population (Kalmus and Fry, 1980) suffers from lifelong problems in perceiving and producing music in the absence of brain injury (Ayotte et al., 2002). Importantly, these problems cannot be explained by hearing loss, intellectual deficiencies, or lack of music exposure. This developmental disorder is known as congenital amusia (Peretz, 2001) (“amusics” hereafter), and the core deficit lies in musical pitch processing (Foxton et al., 2004; Hyde and Peretz, 2004).

Accumulating evidence suggests that amusics may also have behavioral difficulties with linguistic tone processing (Patel et al., 2008; Nan et al., 2010; Tillmann et al., 2011; Liu et al., 2012; Yang et al., 2013). This condition applies to both speech intonation and lexical tones and can affect the amusics either at the group level (Jiang et al., 2012; Liu et al., 2012) or only for a subgroup of individuals. For example, Patel et al. (2008) found that approximately 30% of amusics among non-tone language speakers had impairments in processing speech intonation, which is used to discriminate questions and statements (Patel et al., 2008). Similarly, our recent research shows that only a subgroup of amusics demonstrated deficits in lexical tone perception (Nan et al., 2010; Yang et al., 2013).

These data suggest that although the linguistic tone deficits in amusics were generally first observed at the group level, it is essential to be aware of the possibility that subgroup differences may exist. This is especially important for follow-up research on amusics' related speech tone difficulties. For instance, a recent study found that Mandarin-speaking amusics were impaired with regard to the categorical perception (CP) of lexical tones (Jiang et al., 2012). It should be noted that CP reflects some of the fundamental aspects of speech processing (Harnad, 1987). However, behavioral lexical tone difficulties in these amusics were not reported, nor were the possible subgroup differences in behavioral lexical tone perception. It is thus unclear whether the observed CP impairment applies to all Mandarin-speaking amusics or only to a subgroup of amusics who might have behavioral lexical tone difficulties.

The current study thus aimed to investigate the categorical nature of lexical tone perception in Mandarin-speaking amusics with and without behavioral lexical tone deficits. In addition to taking the subgroup differences into account, we also carefully chose an appropriate step size for the CP measurement. In the previous CP study with amusics (Jiang et al., 2012), the step size of the discrimination pairs used was a 6-Hz difference, which fell in the normal range of the just-noticeable differences (JNDs) for F0 contour discrimination among normal Mandarin listeners (i.e., 4–8 Hz) (Liu, 2013). This 6-Hz step size might not be large enough to reveal the categorical nature of tone perception in the amusics given their congenital pitch impairments. We thus used a relatively larger step size (20 Hz) to minimize the possible confounding effect of the step size due to amusics' pitch deficits. Moreover, stimulus duration was included as an independent variable (at two levels: 100 and 200 ms) to examine its effect upon the CP of lexical tones in amusics. It has been suggested that a lengthened syllable duration significantly enhances the pitch contour identification performance among native speakers of both tone and non-tone languages (Blicher et al., 1990).

Overall, it was expected that only amusics with difficulties identifying and discriminating lexical tones would exhibit impaired CP of lexical tones, while amusics without these problems would perform the two tasks as well as the normal controls. Research into the duration effect on CP could also provide implications for improving tone perception performance in amusics.

## Methods

### Listeners

Three groups of listeners participated in the current study: normal controls, amusics without tone agnosia (hereafter, pure amusics), and amusics with tone agnosia (hereafter, tone agnosics). The control group consisted of 10 participants (4 females and 6 males), while there were 12 listeners in the pure amusic group (6 females and 6 males), and six listeners (2 females and 4 males) in the tone agnosic group. All participants were native speakers of Mandarin Chinese. They were all right-handed according to the Edinburgh Handedness Inventory (Oldfield, 1971). Each participant had normal hearing sensitivity, with thresholds equal to or below 20 dB HL at octave intervals between 250 and 8000 Hz in both ears, as measured by pure-tone audiometry (ANSI, 2010). The experiment was approved by the ethics review board at Beijing Normal University. All participants were paid for their participation. None of the participants had received extracurricular music training. The characteristics of these three groups are shown in Table 1.

**Table 1:** **Characteristics of the control, pure amusic, and tone agnosic groups and the percentage scores of the MBEA and lexical tone perception tests**.

	**Controls (*n* = 10)**	**Pure amusics (*n* = 12)**	**Tone agnosics (*n* = 6)**
Mean age (range)	24.0 (22–26)	23.1 (19–26)	23.83 (21–31)
Male/female	6/4	6/6	4/2
Handedness right/left	10/0	12/0	6/0
PIQ (SD)^*^	121.7 (10.2)	117.6 (6.2)	112.7 (9.2)
VIQ (SD)^*^	129.8 (9.2)	123.7 (6.2)	126.2 (4.9)
**MBEA MEAN (SD)**			
Scale	85.5 (10.5)	65.3 (12.1)	64.2 (6.8)
Contour	86.5 (6.8)	68.6 (7.0)	65.3 (5.6)
Interval	83.1 (10.0)	59.9 (12.3)	59.8 (8.2)
Rhythm	89.8 (6.9)	65.9 (13.9)	68.0 (9.2)
Meter	78.3 (19.8)	56.8 (14.0)	58.5 (12.4)
Memory	94.5 (4.7)	72.0 (10.3)	69.7 (18.3)
Global	86.2 (5.3)	64.7 (4.6)	64.3 (5.9)
**LEXICAL TONE MEAN (SD)**			
Discrimination	96.8 (4.1)	93.9 (8.1)	75.3 (9.4)
Identification	96.8 (4.4)	94.5 (5.1)	57.0 (19.9)
Global	96.8 (3.9)	94.6 (4.6)	65.5 (14.2)

* *PIQ, performance intelligence quotient; VIQ, verbal intelligence quotient. SD indicates standard deviation*.

Each participant was screened by two tests before the experiments of categorical tone perception: the Montreal Battery of Evaluation of Amusia (MBEA) (Peretz et al., 2003) and the lexical tone tests used in our previous study (Nan et al., 2010). The MBEA includes six subtests: three pitch-based tests (scale, contour, and interval), two time-based tests (rhythm and meter), and one memory test. All participants with congenital amusia scored below the cut-off score of 71.7%, corresponding to 2 SDs below the mean of the controls as obtained in our previous study (Nan et al., 2010). The lexical tone tests include lexical tone identification and discrimination tasks. The stimuli in both tasks were naturally spoken monosyllables or bisyllables. It is noteworthy that these tone tasks did not test CP of lexical tones as such. Those who not only met the amusia criterion but also scored less than 80% for the mean accuracy in the lexical tone tests, which corresponded to 3 SDs below the mean of the controls according to our earlier study (Nan et al., 2010), were identified as tone agnosics. These 12 pure amusics and six tone agnosics were screened from approximately 500 normal-hearing participants because the prevalence rates of amusia (4%; Kalmus and Fry, 1980) and tone agnosia (1%; Nan et al., 2010) were quite low.

### Stimulus generation and presentation

**Figure 1:**
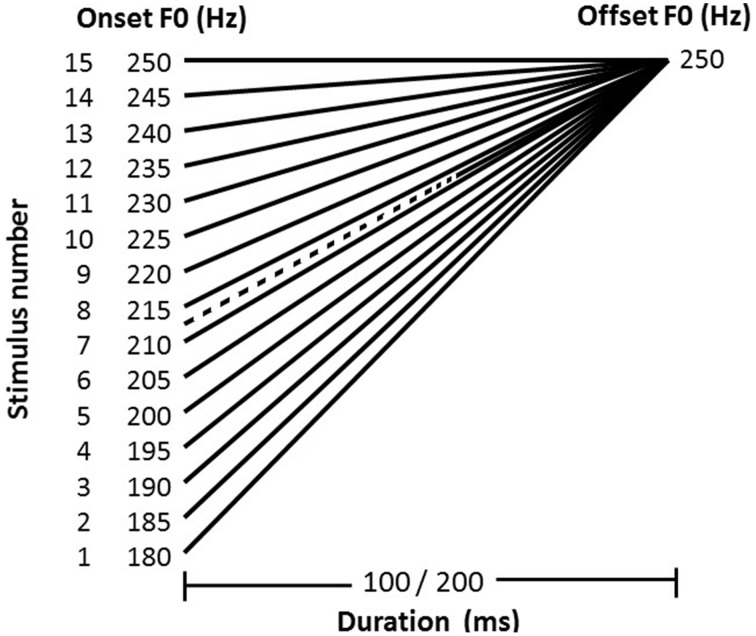
**The F0 contours of the tone 1-2 (rising-to-level) continuum (the solid lines)**. The onset F0 varied from 180 to 250 Hz, with a step size of 5 Hz. The offset F0 was fixed at 250 Hz. The stimulus duration was 100 or 200 ms. The dashed line represents the average tone boundaries among the controls, the pure amusics and the tone agnosics and across both duration conditions (onset F0 stands at about 213 Hz).

**Figure 2:**
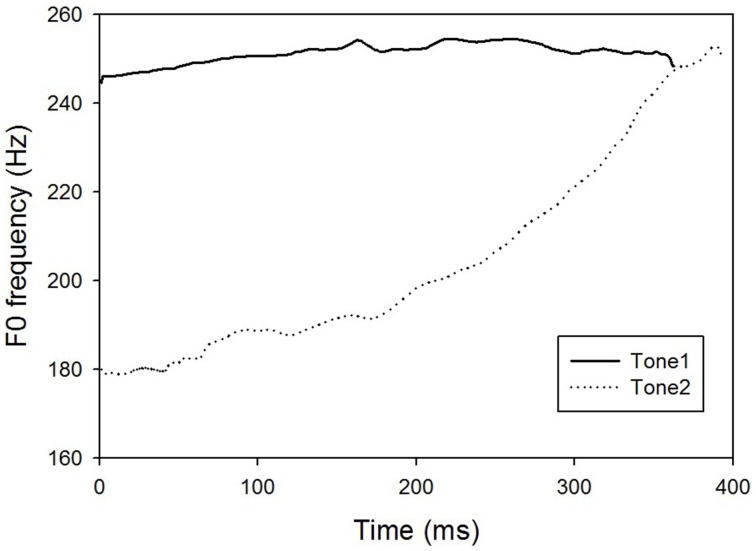
**Time by frequency representations of the stylized F0 contours of tone 1 and tone 2 of vowel /a/**. The solid line and dotted line indicates tone 1 and tone 2, respectively. As can be seen in the figure, the F0 of tone 1 is steady on the high level while the F0 of tone 2 starts on a relatively low level and ascend gradually and ends at high level that is comparable to tone 1.

An isolated Mandarin Chinese vowel /a/ was initially recorded from a young female native Mandarin speaker with a high level tone (tone 1). The carrier vowel /a/ has different lexical meanings for tone 1 and tone 2 in Mandarin Chinese, however, the lexical meanings may have little effect on the CP of Mandarin tones for Mandarin Chinese-native listeners (Liu, 2014). Liu (2014) measured CP of Mandarin tones for three types of stimuli: Chinese vowel /a/, English vowel /ε/, and tone glide (non-speech). Results showed that the slopes and boundaries of CP functions of tone 1 and 2 were not significantly different across the three types of stimuli, indicating that lexical meanings in the stimuli may not affect CP for Mandarin-native speakers with normal hearing. The duration of the original vowel /a/ was 363 ms and the center 100- and 200-ms segments were selected as the standard stimuli for F0 contour manipulation. The F0 contour of the standard stimulus (100- or 200-ms) was replaced by a set of linear F0 contours using a high-fidelity speech synthesizer, STRAIGHT (Kawahara et al., 1999), while preserving the other acoustic features (e.g., spectrogram). By manipulating the onset F0 systematically from 180 to 250 Hz with the offset F0 fixed at 250 Hz, a rising-to-level continuum (see Figure 1; also see the stylized F0 contours of tone 1 and tone 2 of vowel /a/ in Figure 2) consisting of 15 stimuli with an equal step size of 5 Hz was generated. Thus, there were two sets of F0 continua: 100 and 200 ms.

The stimuli were sampled at 48,828 Hz and presented at a 70 dB sound pressure level to the right ear of all participants through SONY MDR-7506 headphones. A Tucker-Davis Technologies (TDT, Alachua, FL) mobile digital sound processor (RM1) was used for signal presentation. The sound pressure levels of the signals were calibrated in an AEC201-A IEC 60319-1 ear simulator by a Larson-Davis sound-level meter (Model 2800) with the linear weighting band. The experimental procedure was run with Sykofizx® v 2.0 software.

### Procedures

Each participant completed two separate tasks in a quiet room: tone identification and discrimination. The two tasks were presented in a counterbalanced order across participants.

#### Measures of tone identification

In the identification task, participants were asked to identify a given stimulus as tone 1 or tone 2. Each participant gave their responses in front of a computer screen by clicking the corresponding button labeled “tone 1” or “tone 2.” For each trial, participants were required to respond within 10 s after the stimulus presentation. The next stimulus was presented automatically 1 s after a response was received. Each stimulus of the F0 continuum was presented 20 times to each participant such that there were 300 trials presented in a random order in one block. Listeners completed two blocks of tone identification: 100 and 200 ms each, with the order of the two duration blocks randomized across listeners.

The tone identification boundary and the tone identification sharpness (i.e., the slope of the identification function) were measured. A logistic model was employed to fit the tone identification function obtained from each participant based on the binomial distribution of the identification scores and the sigmoidal shape of the identification function.

(1) $$p=\frac{1}{1+e^{\frac{-(x-x_{o})}{b}}}$$

In Equation (1), *p* refers to the identification score, *x* refers to the onset F0 frequency of the stimulus, *x*_0_ refers to the identification boundary where the accuracy was 50%, and *b* refers to the sharpness indicator of the identification function. The fitness of the sigmoid model was operated in SigmaPlot® v12.0 that adopted the Marquardt–Levenberg algorithm (Marquardt, 1963) to seek the parameters (*x*_0_ and *b*) of the independent variable (*x*) that give the best fit between the equation and the data (*p*). The algorithm employed an iterative process to determine the best parameters that minimized the sum of the squared differences between the observed and predicted values of the dependent variable (*p*).

#### Measures of tone discrimination

In the discrimination task, 11 pairs were composed by separating two stimuli by 20 Hz (e.g., 180–200 Hz). There were four possible combination forms for each stimulus pair: AA, AB, BA, and BB. The inter-stimulus interval (ISI) was 400 ms. For each trial, after the pair of stimuli were presented, the listener's task was to indicate whether the two stimuli were the same or different by clicking the corresponding button labeled “Same” or “Different” on the computer screen. The response time requirement and stimulus presentation pace were identical to the tone identification task. There were 220 trials (11 pairs × 20 repetitions) presented in a random order in each block, and two blocks (100 and 200 ms each) were completed for each listener. The order of the two duration blocks was also randomized across listeners.

The two experimental tests (tone identification and discrimination) were preceded by a 2-min practice session to familiarize the participants with both tasks. To minimize the training effect, no feedback was given during the practice session.

The peakedness of the discrimination function was measured. As mentioned above, to compute the discrimination score for a given duration, 220 trials were divided into 11 comparison units. Each unit was composed of four types of pairwise comparisons (AA, AB, BA, and BB). The step size of the two different stimuli, A and B, was 20 Hz, significantly higher than the JNDs of Mandarin-native listeners with normal hearing (Liu, 2013). There were 20 trials in each unit. The discrimination score was computed by Equation (2):

(2) p=p("D"|D)×p(D)+p("S"|S)×p(S)

In Equation (2), *p* (“D”|D) and *p* (“S”|S) represent two conditional probabilities, that is, the percentages of “same” (“S”) and “different” (“D”) responses of all of the same (S) and different (D) trials in each comparison unit, while *p* (D) and *p* (S) refer to the probabilities of the different and same pairs among the 20 trials in each unit, respectively.

The discrimination scores for each participant were then computed for the following three measures: the between-category discrimination score (P_bc_), the within-category discrimination score (P_wc_), and the peakedness of the discrimination function (P_pk_). Based on the identification boundary *x*_0_(for example, 212 Hz), the scores for the stimulus pairs across the identification boundary (195–215, 200–220, 205–225, and 210–230 Hz) were averaged and defined as P_bc_, while the average scores of the remaining comparison pairs were coded as P_wc_. P_pk_ was then calculated as the difference between P_bc_ and P_wc_.

## Results

### Tone identification

**Figure 3:**
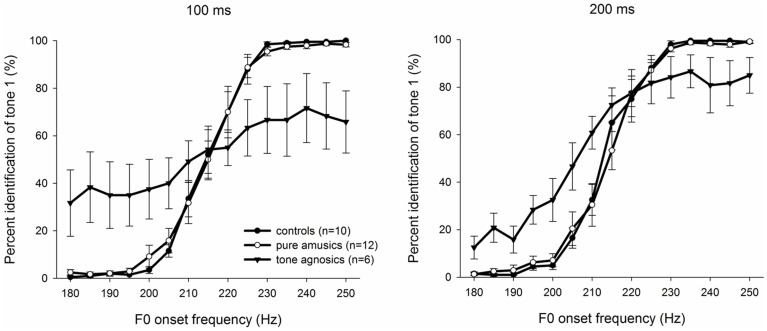
**Identification curves of tone 1 as a function of the onset F0 frequency in the 100-ms condition (left) and the 200-ms condition (right) averaged over participants for each of the three groups of listeners**. Error bars denote standard error of the mean.

As shown in Figure 3, the average percent of stimuli identified as tone 1 is plotted as a function of the onset F0 frequency. The sigmoidal fitting function was computed following the steps introduced above for each signal duration and each group of listeners. The slope of the identification function was then calculated between 30 and 70%, while the tone boundary (*x*_0_) was the F0 frequency with a 50% identification score.

#### Slopes of tone identification functions

The slopes were analyzed in a Two-Way analysis of variance (ANOVA) to determine whether there were any effects on the sharpness of the category boundary, with the listener group (controls, pure amusics, and tone agnosics) as a between-subjects factor and stimulus duration (100 vs. 200 ms) as a within-subjects factor. The results showed a significant effect of the group factor [*F*_(2, 25)_ = 11.293, *p* < 0.05, η^2^ = 0.475, large effect size]. The main effect of duration was not significant [*F*_(1, 25)_ = 0.152, *p* > 0.05], nor was the interaction between group and duration [*F*_(2, 25)_ = 0.620, *p* > 0.05]. Bonferroni-corrected *post hoc* tests indicated that for both stimulus durations, the sharpness of the category boundary of tone agnosics were significantly shallower than those of controls and pure amusics (both *ps* < 0.05), while there was no significant difference between the latter two groups (*p* > 0.05).

#### Tone boundary

Tone boundaries of the three groups in 100- and 200-ms conditions are shown in Table 2. Similarly, a Two-Way ANOVA on tone boundary, *x*_0_, was conducted. The results showed no significant effect of the listener group, stimulus duration, and interaction between the group and stimulus duration (all *ps* > 0.05).

**Table 2:** **Mean tone boundaries of the control, pure amusic, and tone agnosic groups in the 100- and 200-ms conditions**.

**Duration (ms)**	**Controls (*n* = 10)**	**Pure amusics (*n* = 12)**	**Tone agnosics (*n* = 6)**
100 (SD)	215.00 (5.83)	214.64 (6.77)	212.90 (12.62)
200 (SD)	213.64 (5.97)	213.65 (6.38)	209.39 (10.16)

*SD indicates standard deviation*.

### Tone discrimination

**Figure 4:**
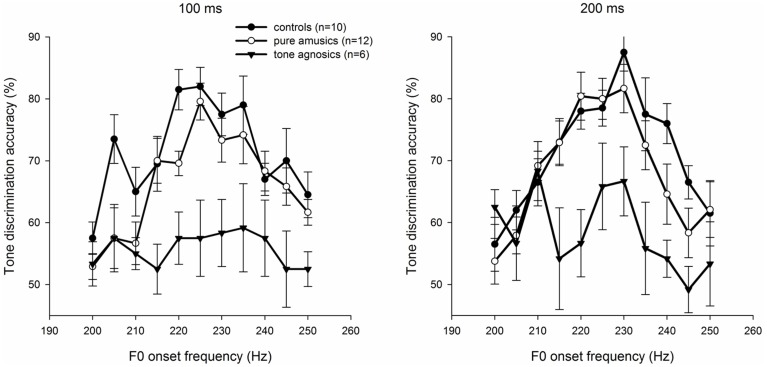
**Tone discrimination scores at 20-Hz as a function of the higher frequency of each pair of tone comparisons for the three groups of listeners with 100-ms (left) and 200-ms (right) stimuli**. Error bars denote standard error of the mean.

Figure 4 illustrates the average discrimination scores pooled across the participants in each group for the 11 comparison units. For example, the discrimination score of 81.5% at 220 Hz of controls for the 100-ms condition means that the control group discriminated the pair of stimuli, 220–200 Hz, with an accuracy of 81.5% at the100-ms duration. Following the method described above, the between- and within-category (P_bc_ and P_wc_, respectively) discrimination scores as well as the value of peakedness (P_pk_) were calculated.

#### Between- and within-category discrimination

As shown in Figure 4, discrimination functions showed similar patterns for the control and pure amusic groups. Participants in both groups had higher scores on the between-category discriminationthan the within-category discrimination, whereas tone agnosics had chance levels for all comparison units.

A Three-Way ANOVA with the listener group as the between-subjects factor and stimulus duration and discrimination location (between- or within-category discrimination; P_bc_ and P_wc_, respectively) as the within-subjects factors for the discrimination scores was conducted. The results indicated significant main effects of the group [*F*_(2, 25)_ = 14.058, *p* < 0.05, η^2^ = 0.529, large effect size] and the discrimination location [*F*_(1, 25)_ = 47.783, *p* < 0.05, η^2^ = 0.551, large effect size], while the duration effect was marginally significant [*F*_(1, 25)_ = 3.904, *p* = 0.059]. The group × discrimination location interaction was also significant [*F*_(2, 25)_ = 5.662, *p* < 0.05, η^2^ = 0.130, medium effect size], whereas all other multi-factor interactions were non-significant (all *ps* > 0.05). As shown in Figure 4, *post hoc* Bonferroni-corrected tests revealed that for both stimulus durations, the between-category performance (P_bc_) of controls and pure amusics was significantly better than the within-category performance (P_wc_; all *ps* < 0.05), indicating the presence of peaks in the discrimination functions for both controls and pure amusics across the two stimulus durations. However, no significant difference was observed between P_bc_ and P_wc_ for the tone agnosic group for either stimulus duration (both *ps* > 0.05), indicating no peak in their discrimination functions. Additionally, regardless of the stimulus duration, tone agnosics performed worse than controls and pure amusics on both between- and within-category scores (all *ps* < 0.05), except for the within-category performance under the 200-ms condition (*p* > 0.05).

#### Peakedness of discrimination

Significant peaks were observed in both the control and the pure amusic groups, but not in the tone agnosic group (see Figure 4). A two-factor (between-subjects factor: listener group, and within-subjects factor: stimulus duration) ANOVA was conducted with peakedness (P_pk_) as the dependent variable. The results showed a significant main effect of the listener group [*F*_(2, 25)_ = 5.637, *p* < 0.05, η^2^ = 0.311, large effect size]. Bonferroni-adjusted multiple comparisons indicated that both controls and pure amusics obtained a significantly higher discrimination peak than tone agnosics (both *ps* < 0.05), while there was no significant difference between the control and the pure amusic groups (*p* > 0.05). Neither stimulus duration nor the interaction between the listener group and the stimulus duration was found to be significant (all *ps* > 0.05; see Figure 4).

## Supplemental test of tone discrimination for tone agnosics

It is important to note that the lack of peaks in the discrimination functions for the tone agnosics, as observed above, might reflect their non-categorical perception of Mandarin tones. Alternatively, it might also be due to their reduced psychophysical capacity, if any, to discriminate tone contour changes across the tone boundary. That is, the 20-Hz difference in each stimulus pair of the discrimination task might not be large enough, e.g., smaller than the JNDs of the tone contour changes of tone agnosics. In a pilot study on the JNDs of tone contour changes for controls, pure amusics and tone agnosics, the tone contour discrimination thresholds of tone agnosics ranged from 20 to 30 Hz, significantly higher than the thresholds of the other two groups and also greater than the step size of 20 Hz used in the current discrimination task. Thus, to rule out the possible confounding effect of psychophysical capacity, an additional tone discrimination task was conducted for the tone agnosic group by changing the step size from 20 to 35 Hz, higher than their JNDs. Five of the six tone agnosics completed this additional discrimination task, the procedure of which was identical to the one described above.

**Figure 5:**
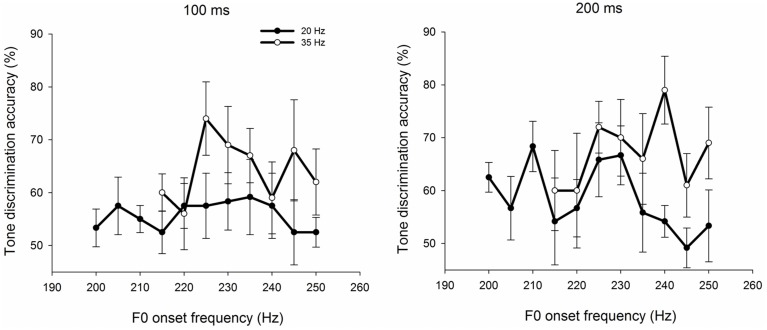
**Tone discrimination scores at 20-Hz and 35-Hz as a function of the higher frequency of each pair of tone comparisons for the tone agnosic group with 100-ms (left) and 200-ms (right) stimuli**. Error bars denote standard error of the mean.

The results showed that the larger step size between the stimulus pairs did not result in any discrimination peaks for tone agnosics (see Figure 5). A Two-Way repeated-measures ANOVA (within-subjects factors: stimulus duration and discrimination location) was run with the discrimination scores as the dependent variable for the tone agnosic group. None of the main and interaction effects were significant (all *p*s > 0.05), indicating no peak in the discrimination functions with the 35-Hz step size (e.g., no significant difference between P_bc_ and P_wc_). These results suggested that, for tone agnosics, the absence of a peak in either discrimination function was more likely due to their lack of CP of lexical tones rather than their reduced psychophysical capacity to discriminate tone contour changes.

## Discussion

Congenital amusia is a developmental disorder that affects the processing of musical pitch (Ayotte et al., 2002; Peretz et al., 2002). Recent evidence suggests that this deficit may extend to speech, affecting the processing of speech prosody (Patel et al., 2008; Liu et al., 2010) and lexical tones (Nan et al., 2010; Tillmann et al., 2011). Some studies indicate that the linguistic tone difficulties might implicate only a subgroup of amusics while sparing the rest (Patel et al., 2008; Nan et al., 2010; Yang et al., 2013). For instance, our earlier studies suggest that there are two subgroups of Mandarin-speaking amusics: one has behavioral lexical tone processing impairments while the other does not (Nan et al., 2010; Yang et al., 2013). The current study aimed to examine the CP of lexical tones in Mandarin-speaking amusics with and without behavioral lexical tone deficits. Our results revealed that only amusics with tone agnosia showed significant impairment in the CP of lexical tones, while the pure amusics perceived lexical tones in a categorical manner similar to that of the controls.

Our results thus refine a recent finding that Mandarin-speaking amusics had CP impairments for lexical tones (Jiang et al., 2012), showing that not all Mandarin-speaking amusics were impaired but only those with behavioral lexical tone difficulties. These data imply that congenital amusia alone does not necessarily result in a deficit in the CP of lexical tones. The impaired CP of lexical tones only implicates those amusics with behavioral lexical tone difficulties, whereas amusics without lexical tone deficits are spared. These findings are consistent with the previous standpoint that the pitch processing deficits in music might not always coexist with those in speech (Nan et al., 2010; Tillmann et al., 2011; Yang et al., 2013). The dissociation between musical pitch and lexical tone deficits corroborates the hypothesis that despite some overlap across music and speech, there might be two different pitch-related systems subserving pitch processing in these two domains (Zatorre and Baum, 2012).

The current study also demonstrates the necessity of taking the behavioral speech tone performance into account and being aware of the possible subgroup differences for any further attempt to understand the linguistic tone deficits related to congenital amusia. As in the current study, if the subgroup differences in behavioral lexical tone difficulties were ignored and the amusics with or without lexical tone deficits were taken together as one whole group of amusics, the results would have found that the combined amusic group performed significantly worse than the controls in both discrimination and identification tasks. Specifically, for the discrimination task, the combined amusic group had significantly smaller category differences (between-category vs. within category performance) than the controls, *F*_(1, 26)_ = 7.314, *p* = 0.012. For the identification task, the combined amusic group demonstrated a significantly shallower identification slope than the controls, *F*_(1, 26)_ = 5.170, *p* = 0.031. It is thus clear that if the possible subgroup difference in behavioral lexical tone deficits were overlooked, the CP impairment would have been wrongly attributed to the whole amusic group instead of the subgroup of amusics who had behavioral lexical tone deficits.

Please note that besides the subgroup differences in behavioral lexical tone deficits, step size might be another factor contributing to the previous finding of the lack of CPs of lexical tones in the amusic group (Jiang et al., 2012). Rather than the 6-Hz step size used in Jiang et al.'s study (2012), which might be too small to reveal the CP of lexical tones in congenital amusics, we employed a larger step size (20-Hz). The results showed that the pure amusics exhibited significantly higher discrimination accuracy for between-category tone pairs than the within-category tone pairs, resulting in clear discrimination peaks across the identification boundary, which was one of the essential characteristics of categorical perception. In contrast, tone agnosics had no discrimination peaks, as predicted by the shallow slopes of the identification function. Importantly, the lack of peaks in tone agnosics was not likely due to a reduced sensitivity to tone pitch change, as the same pattern persisted in tone agnosics at the enlarged step size of 35 Hz.

The stimulus duration was included for the first time in studies of categorical perception. Interestingly, the results showed that the length of stimuli had little effect on the performance of the CP of lexical tones in pure amusics and tone agnosics because both groups exhibited consistency across different stimulus durations (i.e., 100 and 200 ms). Moreover, the pure amusics showed robust CP of lexical tones, which was similar to that of the controls, even when the stimulus duration was relatively short (i.e., 100 ms). In contrast, tone agnosics failed to perceive lexical tones categorically under both duration conditions. Notably, as shown in their psychometric curves, tone agnosics did not reach 0 and 100% in the identification task for either duration, confirming their behavioral lexical tone deficits as suggested by the pre-test.

The observation of the lack of CP for lexical tones in Mandarin-speaking amusics with behavioral tone deficits raises new research questions regarding the relationship between the CP of lexical tones and tone language experiences. Previous studies comparing tonal and non-tonal language speakers with the CP paradigm came to a general conclusion that only those who spoke tone languages showed classic CP for lexical tones, while individuals without tone language experience perceived tones on a psychophysical basis (Wang, 1976; Francis et al., 2003; Xu et al., 2006; Peng et al., 2010; Liu, 2013). Interestingly, despite being exposed to the tone language environment since birth, tone agnosics could not successfully produce a peak for the between-category discrimination and exhibited a shallow slope of tone identification functions, showing a lack of CP for lexical tones similar to non-tonal language speakers (Wang, 1976; Francis et al., 2003; Xu et al., 2006; Peng et al., 2010; Liu, 2013). Our results thus suggest that tone language experience does not necessarily guarantee the CP of lexical tones.

## Application and future research

Despite the common deficits in musical pitch processing, the pure amusics and the tone agnosics are different in terms of lexical tone pitch processing, as shown in the current study as well as in previous research (Nan et al., 2010; Tillmann et al., 2011; Yang et al., 2013). Note that the non-categorical perception of lexical tones may reflect the impairment of fundamental aspects of speech tone processing, future studies should further investigate the mechanisms of lexical tone deficits in tone agnosics to help elucidate the relations and interactions between music and speech perception. In addition, it would be interesting to determine whether the pure amusics who seem to perform well in various types of behavioral tone tests (e.g., Nan et al., 2010) also have similar integrity from a neuroimaging perspective. Furthermore, it is vital to understand how the currently observed lexical tone deficits might affect the learning of a tone language. As shown in a recent mismatch negativity research, Mandarin speakers exhibited fast learning effects for novel tone-segment combinations (Yue et al., 2014). Future research is warranted to examine whether the amusic individuals with lexical tone deficits would be able to demonstrate a similar learning pattern.

## Summary

Whether having difficulty in lexical tone perception separates pure amusics and tone agnosics. The current study investigated lexical tone perception in congenital amusics with the traditional CP paradigm. The CP of lexical tones was intact in pure amusics but impaired in tone agnosics. These results reconcile the previous debate on whether tone pitch processing is compromised in congenital amusics. That is, pitch processing deficits in music do stretch into speech, but only a small proportion of this special group is affected and suffers from tone perception deficits. Overall, the present findings improve our knowledge about the lexical tone processing abilities in congenital amusics. The different performance between congenital amusics with and without tone agnosia has provided a new perspective on the relationship between music and speech perception.

### Conflict of interest statement

The authors declare that the research was conducted in the absence of any commercial or financial relationships that could be construed as a potential conflict of interest.
